# Mobile App for Mental Health Monitoring and Clinical Outreach in Veterans: Mixed Methods Feasibility and Acceptability Study

**DOI:** 10.2196/15506

**Published:** 2020-08-11

**Authors:** Lisa M Betthauser, Kelly A Stearns-Yoder, Suzanne McGarity, Victoria Smith, Skyler Place, Lisa A Brenner

**Affiliations:** 1 Veterans Affairs Rocky Mountain Mental Illness Research Education and Clinical Center Aurora, CO United States; 2 Department of Physical Medicine and Rehabilitation School of Medicine University of Colorado Aurora, CO United States; 3 Cogito Corporation Boston, MA United States; 4 Departments of Psychiatry and Neurology School of Medicine University of Colorado Aurora, CO United States

**Keywords:** veterans, mobile app, smartphone, mental health, acceptability, feasibility

## Abstract

**Background:**

Advances in mobile health (mHealth) technology have made it possible for patients and health care providers to monitor and track behavioral health symptoms in real time. Ideally, mHealth apps include both passive and interactive monitoring and demonstrate high levels of patient engagement. Digital phenotyping, the measurement of individual technology usage, provides insight into individual behaviors associated with mental health.

**Objective:**

Researchers at a Veterans Affairs Medical Center and Cogito Corporation sought to explore the feasibility and acceptability of an mHealth app, the Cogito Companion.

**Methods:**

A mixed methodological approach was used to investigate the feasibility and acceptability of the app. Veterans completed clinical interviews and self-report measures, at baseline and at a 3-month follow-up. During the data collection period, participants were provided access to the Cogito Companion smartphone app. The mobile app gathered passive and active behavioral health indicators. Data collected (eg, vocal features and digital phenotyping of everyday social signals) are analyzed in real time. Passive data collected include location via global positioning system (GPS), phone calls, and SMS text message metadata. Four primary model scores were identified as being predictive of the presence or absence of depression or posttraumatic stress disorder (PTSD). Veterans Affairs clinicians monitored a provider dashboard and conducted clinical outreach when indicated.

**Results:**

Findings suggest that use of the Cogito Companion app was feasible and acceptable. Veterans (n=83) were interested in and used the app; however, active use declined over time. Nonetheless, data were passively collected, and outreach occurred throughout the study period. On the Client Satisfaction Questionnaire–8, 79% (53/67) of the sample reported scores demonstrating acceptability of the app (mean 26.2, SD 4.3). Many veterans reported liking specific app features (day-to-day monitoring) and the sense of connection they felt with the study clinicians who conducted outreach. Only a small percentage (4/67, 6%) reported concerns regarding personal privacy.

**Conclusions:**

Feasibility and acceptability of the Cogito Corporation platform to monitor mental health symptoms, behaviors, and facilitate follow-up in a sample of veterans were supported. Clinically, platforms such as the Cogito Companion system may serve as useful methods to promote monitoring, thereby facilitating early identification of risk and mitigating negative psychiatric outcomes, such as suicide.

## Introduction

Mental health disorders are common, with 46.6 million United States (US) adults using mental health services in 2017 [1]. Approximately 2.1 million US individuals received Veterans Affairs mental health care between 2006 and 2010 [2]. Although not all veterans seek care at Veterans Health Administration facilities [3], one recent study [4], using data from the 2012-2014 National Survey on Drug Use and Health, found that 34% of veterans with any mental illness had received at least one form of mental health treatment in the preceding 12 months. Veterans commonly experience symptoms, such as those associated with posttraumatic stress, mood, and anxiety disorders. In the past, these symptoms have been monitored using self-report measures, which can yield unreliable and incomplete information [5].

Advances in smartphone technology, such as mobile health (mHealth) apps, provide the opportunity for continuous monitoring and tracking of mental health symptoms and behaviors [6-8]. The majority of individuals living in the US own and use smartphones [9], which increases the feasibility of using this technology to detect behavior change; mHealth apps which employ methods such as digital phenotyping can assist patients in adhering to treatment via self-monitoring, as well as assist health care providers in monitoring symptom progression outside of clinical visits. Ideally, such monitoring would occur in real time [7], and include subjective self-report of mental health symptoms, as well as objective behavioral data drawn from passive monitoring of an individual’s smartphone. Such behavioral data may be used to observe and track changes in functioning (eg, changes in social connectivity as indicated by text messaging patterns). Clinical outreach can then be initiated. Although the Department of Defense and Veterans Affairs have created and distributed mHealth apps for service members and veterans [10], these apps predominantly provide psychoeducation or facilitate coping strategies, but do not allow patients and health care providers to interactively track symptoms and behaviors in real time.

Cogito Corporation, in conjunction with the Defense Advance Research Project Agency of the Department of Defense, developed a secure, federally compliant mHealth smartphone app, the Cogito Companion, to collect and analyze passive data from the personal smartphones of participants. Data collected (eg, vocal features and digital phenotyping of everyday social signals) are analyzed in real time. Passive data collected include location via global positioning system (GPS) information, phone calls, and SMS text message metadata (including counts and frequency [11]). These data were transformed in Cogito’s proprietary algorithms to provide scores of behavior health indicators that can be shared directly with patients in the mobile app and with providers in real time via a clinical dashboard [11].

Initial data sets (civilians and veterans experiencing at least one symptom of depression or posttraumatic stress disorder) were used to create Cogito’s proprietary algorithms. Four primary model scores were identified as being predictive of the presence or absence of depression or posttraumatic stress disorder based on DSM-IV symptom criteria and included *mood* (indicative of a depressed mood almost every day), *out-and-about* (indicative of diminished interest or pleasure in activities), *socially connected* (indicative of avoidance of activities, places, and people), and *energized* (indicative of fatigue or loss of energy). Model scores ranged from 0 to 100, where a score of 100 indicated a low probability of the presence of the symptom, and 0 indicated a high probability of the presence of the symptom. While initial validity of the model scores and of the feasibility of implementing of the Cogito Companion system were demonstrated, further investigation of acceptability of this app to facilitate symptom monitoring in veterans was suggested [11].

The objective of this study was to evaluate whether veterans would be willing to download and use the app thereby allowing clinicians to monitor output from the app and provide outreach, if indicated. Such acceptability and feasibility data would yield important information for future implementation or efficacy studies in this population.

## Methods

### Study Design

A mixed methodological approach was used to test the feasibility and acceptability of the Cogito Companion system. Acceptability was defined as the perceived suitability of the intervention [12] and was measured by completion of a satisfaction questionnaire and with data from structured qualitative interviews. Acceptability was determined using a criterion defined a priori—more than 70% of the sample providing a score of 24 or higher on the Client Satisfaction Questionnaire–8 and responses to structure qualitative interviews. Feasibility [12] was defined as the ease of implementation of the app among veterans. Feasibility was descriptively measured based on ease of recruitment, rate of enrollment, and rate of attrition; and frequency of clinical outreach calls based on Cogito Companion model scores and responses to a self-reported suicide risk item on biweekly mental health surveys. Veterans’ use of the app was measured by in-app usage metrics and completion of a post-app user experience questionnaire.

### Participants

Participants were US military veterans receiving or eligible to receive care at a mountain state Veterans Affairs Medical Center. Veterans were included if they were between the ages of 18 and 89, had an Android-based smartphone, were willing to use their personal data plan during the study, and were able to provide informed consent. Participants were excluded if they were participating in a conflicting interventional study at the local Veterans Affairs Medical Center. Participants were compensated for the baseline and follow-up visits and were provided monthly payments to offset smartphone data costs.

### Procedures

Colorado Multiple Institutional Review Board approval was obtained. Data collection occurred between October 2016 and September 2018. During the baseline assessment, veterans completed clinical interviews and self-report questionnaires. Study personnel also provided instructions on how to download the Cogito Companion app and answered any questions that arose during the download process. Participants were provided with an app user guide and were informed that the app would collect behavioral information from their smartphone to evaluate their mood, level of stress, and general well-being. They were told they could interact with the app by monitoring their own scores and by leaving audio recordings. App interaction also occurred via biweekly mental health surveys. A notification was displayed by the app when surveys became available. Resources regarding mental health, well-being, and safety were also embedded within the app. After 3 months, participants were contacted to complete an in-person or over-the-phone follow-up assessment and were instructed to uninstall the app. Follow-up assessments included clinical interviews and self-report questionnaires. Data from full measures are available upon request, as not all data is presented in this paper.

### Measures and Materials

#### Clinical Interviews and Assessments

The Structured Clinical Interview for DSM-5 Disorders-Research Version [13] was used to assess current and lifetime psychiatric diagnoses. The Ohio State University Traumatic Brain Injury Identification Method [14], a valid and reliable structured clinical interview procedure, was used to obtain lifetime history of traumatic brain injury [14,15]. The Narrative Evaluation of Intervention Interview [16], a structured qualitative interview, was used to obtain feedback regarding the intervention. The Patient Health Questionnaire–9 [17], a 9-item self-report module from the Primary Care Evaluation of Mental Disorders diagnostic instrument, was used every 2 weeks to assess and monitor symptoms of depression: item 9 (ie, thoughts of being better off dead) was used to monitor ongoing suicide risk. The Client Satisfaction Questionnaire–8 [18], an 8-item questionnaire, was used to assess patient satisfaction with the intervention. Cogito’s Technology Assessment Survey was used to evaluate how likely participants would be to share data from this app with others, overall privacy perceptions, and the degree to which behaviors may have changed as a result of app use.

#### Cogito Companion App

**Figure 1:**
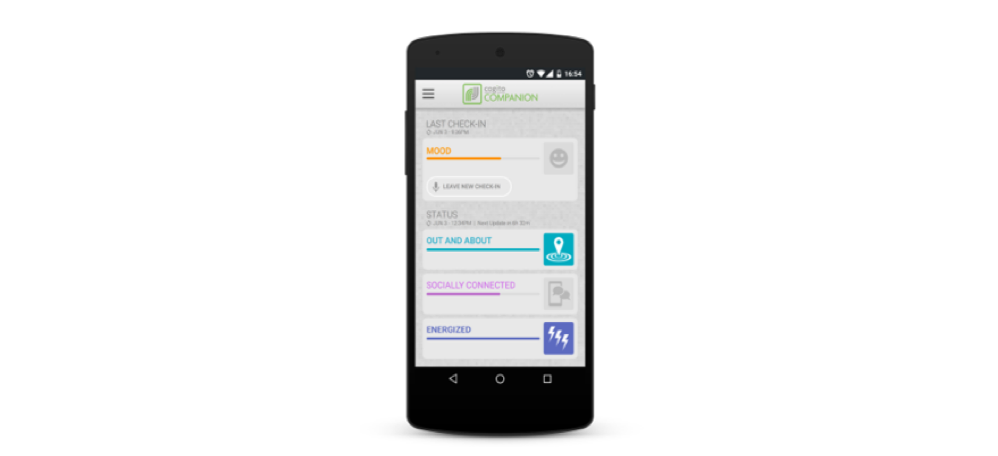
Cogito Companion app.

Once downloaded, the Cogito Companion app (see Figure 1) was able to continuously collect passive data [11]. Model scores generated by the passive data were displayed daily after the first 7 days postdownload. Model scores for audio check-ins were available immediately after the recording was completed. These scores were also presented in a clinician dashboard for the study team to monitor. Clinical outreach occurred based on 3 primary model scores—socially connected, out-and-about, and energized—or if a response to biweekly Patient Health Questionnaire–9 item 9 (ie, thoughts of being better off dead) was a score of 2 or greater. Clinical outreach facilitated treatment with mental health providers within the Veterans Affairs Medical Center, as needed, if the veteran appeared to be at increased risk for suicide or struggling with risk factors associated with suicide risk. The audio check-in score was not used for clinical monitoring purposes.

### Data Analysis

#### Quantitative Analysis

Sample characteristics and descriptive analyses were performed using SAS software (version 9.4; SAS Institute). Data are presented as counts, percentages, median, and range.

#### Qualitative Analysis

Qualitative interviews were analyzed for all participants who completed the 3-month follow-up visit. Investigators used a thematic analysis method [19] rooted in the qualitative descriptive approach [20]. Thematic analysis included six steps: (1) familiarization with the data by reading transcripts, (2) generation of initial codes, (3) identification of themes, (4) review of themes, (5) refinement of the themes, and (6) selection of quotes and examples. We ensured that data saturation [21] occurred, that is, that no new themes or new experiences with the app were noted. Furthermore, data adequacy [22] was achieved; themes that emerged were comprehensive and little to no variation was observed across interviews. All salient themes are presented below.

Scientific rigor was facilitated through a step-by-step approach to examining the interviews. Three investigators (LMB, KSY, SM), who were not affiliated with Cogito Companion, noted and shared their biases at the start of their coding process. At least two investigators reviewed each interview, noting themes, impressions and descriptive quotes on previously constructed coding sheets based on the structure of the qualitative interview (ie, description and evaluation of the app) [16]. Investigators met to review, document, and achieve consensus regarding common themes and identify illustrative quotes. Descriptive summaries of commonly observed themes were generated [20,23]. Themes of benefits, motivation to use the app, barriers experienced using the app, and general use of the app were noted.

## Results

### Baseline Sample Characteristics

Sample characteristics for the participants (N=83) are displayed in Table 1. Individuals in the sample were predominantly men (72/83, 86.7%), white (52/83, 62.7%), and had served in the Army (46/83, 55.4%).

**Table 1:** Baseline characteristics of the sample.

Participant characteristics (N=83)		Value
Age (years), median (range^a^)		50 (24-76)
**Gender, n (%)**		
	Male	72 (87)
	Female	11 (13)
**Race, n (%)**		
	White	52 (63)
	Black or African American	16 (19)
	Multiracial	5 (6)
	Other	10 (12)
**Military branch, n (%)**		
	Army	46 (55)
	Air Force	12 (15)
	Navy	8 (10)
	Marines	16 (19)
	Multiple branches	1 (1)
**Rank at separation, n (%)**		
	Enlisted	57 (69)
	Noncommissioned officer	20 (24)
	Officer	6 (7)
**Deployed, n (%)**		
	No	30 (36)
	Yes	53 (64)
**Combat experience, n (%)**		
	No	42 (51)
	Yes	41 (49)
**Marital status, n (%)**		
	Married	29 (35)
	Single	20 (24)
	Cohabitating	4 (5)
	Widowed	4 (5)
	Divorced or separated	26 (31)
**Employment status, n (%)**		
	Full-time	23 (28)
	Part-time	7 (8)
	Unemployed	30 (36)
	Retired	22 (27)
	Missing	1 (1)
**Psychological disorders^b^, n (%)**		
	Alcohol abuse	12 (15)
	Major depressive disorder	14 (17)
	Posttraumatic stress disorder	20 (25)
	Substance abuse	9 (11)
	Missing^c^/none	28 (34)
**History of TBI, n (%)**		
	Mild only	48 (58)
	Moderate to severe only	4 (5)
	Mild and moderate to severe	9 (11)
	None	22 (26)
	Missing	1 (1)

^a^Minimum to maximum.

^b^N=82.

^c^Data for 1 individual was missing.

### Feasibility

**Figure 2:**
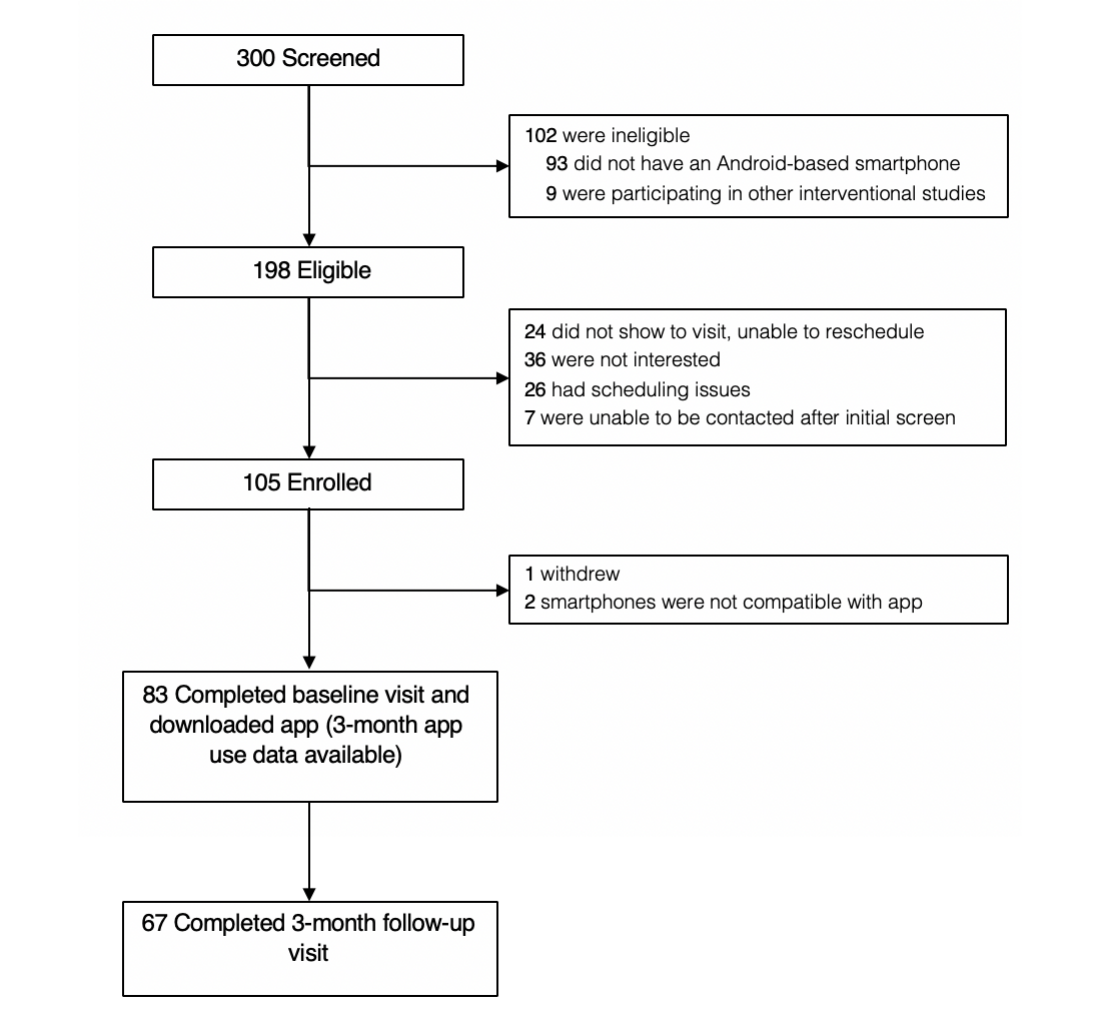
Recruitment flow diagram.

**Figure 3:**
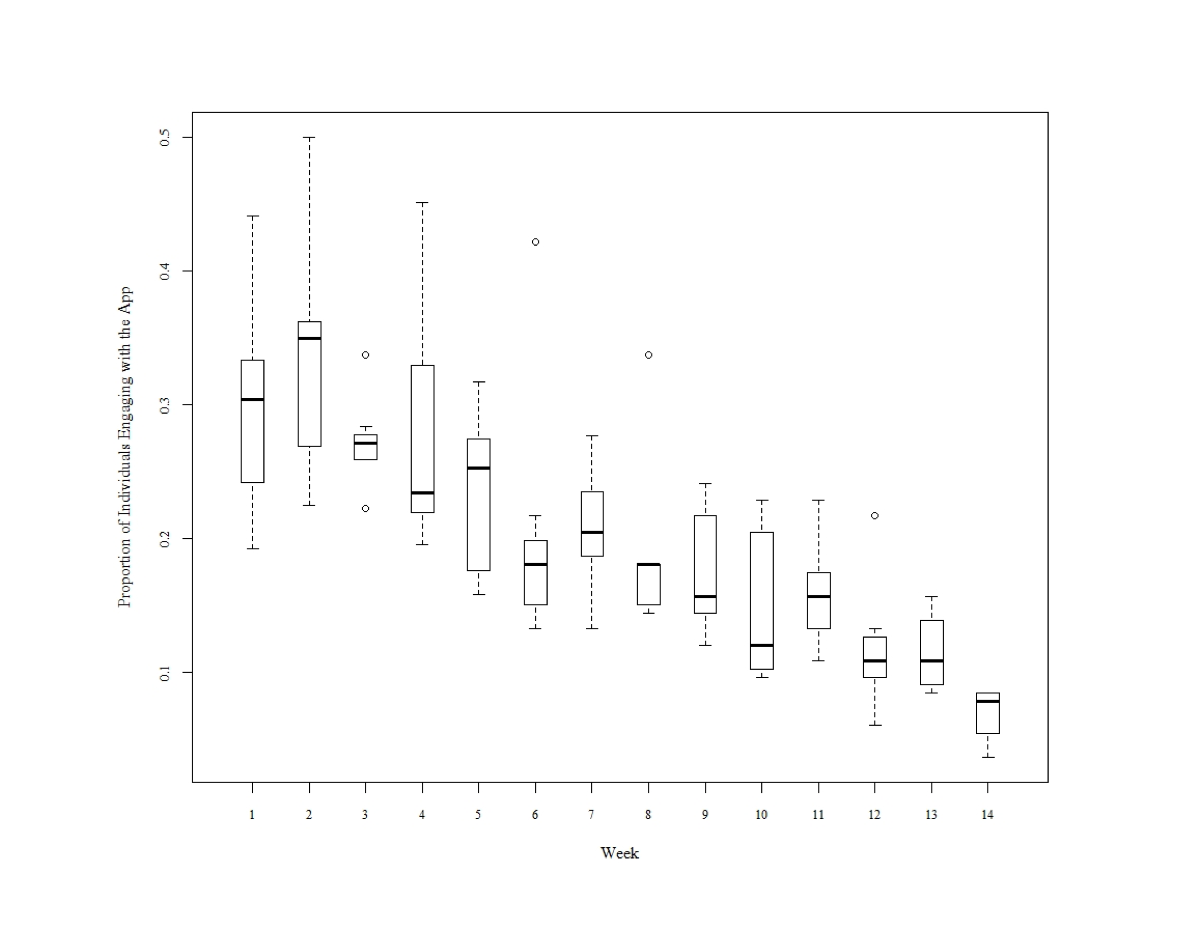
Cogito Companion app engagement. Note: Active app use data is incomplete for the first 7 participants. All available data is displayed.

A flowchart of study eligibility, enrollment, and completion is presented in Figure 2. Although many veterans expressed an interest in the study, about one-third (102/300, 34%) were ineligible because of smartphone or data plan related issues (eg, iPhone versus Android-based smartphone). Scheduling issues and difficulty contacting veterans had an impact on enrollment. Approximately 18% (36/198) of veterans who were eligible were not interested in participating; reasons for this were not collected. No feasibility issues regarding the app download process were observed. Veterans who participated in the 3-month follow-up (67/83, 81%) were asked to subjectively rate the frequency of their app use. These data are shown in Table 2. App interaction data over the course of the study (approximately 90 days) are shown in Figure 3.

**Table 2:** Cogito Companion app use over the 3-month study period.

App action		Number of participants (N=83), n (%)	Number of times, median (range^a^)
Clicked on the home screen		83 (100)	24 (1-191)
Clicked on the help screen		62 (75)	1 (1-9)
Clicked on a daily model score		81 (98)	9 (1-49)
Clicked on a survey		74 (89)	4 (1-41)
Clicked on an audio recording		74 (89)	6.5 (1-83)
First biweekly PHQ-9^b^ survey completed		54 (65)	N/A^c^
Last biweekly PHQ-9 survey completed		39 (47)	N/A
**How often did you use the app?^d^**			
	Never	1 (2)	N/A
	Occasionally	17 (25)	N/A
	Moderately	24 (36)	N/A
	Frequently/often	25 (37)	N/A

^a^Minimum to maximum.

^b^PHQ-9: Patient Health Questionnaire–9.

^c^N/A: not applicable.

^d^Question was administered at follow-up approximately 3 months postdownload. N=67.

A potential limitation of app use was participant misunderstanding about which smartphone behaviors contributed to their model scores, though it is unclear whether this had an impact on app use. A veteran commented:

...the out-and-about and socially connected, I wasn’t sure about those two...I wasn’t comfortable with [the scores] only because I didn’t know.

Some participants noted that they did not know how frequently they should be answering surveys, leaving audio check-ins, and interacting with the app. Many participants recommended having additional notifications to complete surveys, complete check-ins, and for in-app feedback. Furthermore, some veterans believed scores could be intentionally influenced. With respect to the mood score derived in part from the audio check-in, one veteran stated:

...so if I try to sound like I’m in a good mood, like with a smile on my face...it can be manipulated.

Technology-related issues emerged as additional barriers to using the app. For example, challenges with wireless internet connectivity disrupted functionality (eg, delayed data transfer resulted in model scores not being calculated or updated). Some participants noted that the app was “buggy,” and scores were “erratic” which may have been due to limited connectivity, lack of SMS text messaging, or disabled GPS functions. Nonclinical study team members contacted 40 veterans to address these technological issues, with 40% (16/40) reporting resolution with one outreach. A system upgrade that disabled survey and audio check-in notifications temporarily had an impact on the participation of 15% (6/40) veterans.

Clinical team members contacted 42% (35/83) veterans over the course of the study either because of their item-9 response on the Patient Health Questionnaire–9 or because of an observable change in the model scores on the clinical dashboard. Approximately 26% (9/35) of the veterans who were contacted by clinical team members had endorsed suicidal ideation on the Patient Health Questionnaire–9. Clinical team members conducted risk assessments, reviewed safety plans, and facilitated outreach to the veteran’s primary mental health care provider. Approximately 30% (10/35) of the clinical contacts were due to observable changes in the model scores; 17% (6/35) reported experiencing increase in posttraumatic stress disorder symptoms or reported psychosocial stressors such as difficulty finding employment or difficulties in relationships, approximately 25% (9/35) veterans reported feeling “fine” and “great,” and the remainder (5/35, 14%) reported physical health issues (eg, colds, postoperative recovery status, or pain) impacting changes in their activities and use of their phone. The remaining clinical contacts (15/35, 43%) were because of missing scores. In such cases, participants required further operational support such as password resets, or instructions to turn on notifications or allowances specific to the app (eg, GPS).

### Acceptability

Results from the Client Satisfaction Questionnaire–8 suggest that 79% (53/67) of the participants found the app intervention acceptable, a score of 24 or above (mean 26.2, SD 4.3). Data from the Cogito Technology Assessment Survey suggest that about half of veterans (34/67, 51%) enjoyed using the app a lot, 45% (30/67) enjoyed using the app a little, and 4% (3/67) did not enjoy using the app at all. Many described the convenience and accessibility of using the app, noting:

I could just do it at my own leisure.

...it was easily accessible...

It was on your phone, you carry your phone everywhere, that was the biggest thing.

More than half (35/67, 52%) of the veterans reported liking the audio check-in feature; 34% (23/67) reported liking the feature of reviewing past scores on the Cogito Technology Assessment Survey. Veterans identified these features as useful “tool[s]” for monitoring daily activity and stress and building self-awareness. A veteran stated:

It made me monitor my stress level, my attitude...monitored my stress and my everyday activities and everything that I do.

Many participants identified the audio check-in feature of the app as the primary way they monitored their day. One participant said:

It made me conscientious about how I’m feeling.

Several veterans referred to the audio check-in as a “diary,” with one veteran stating:

Describing my day, I guess it gave me maybe a rehash of the day and I was able to kind of work through the bad days and stuff, and even on good days I’d even say “hey, it was a good day!” Cool you can have good days.

Veterans commented that the audio check-in feature allowed them to “vent” about their day, without concern of others listening to their expressed thoughts.

Approximately 33% (22/67) of the participant liked the out-and-about app feature. One veteran used these scores to help create change:

...location, moving around and like how physical you are. Coming into contact with others…I was a person that didn’t want to be bothered with nobody...I liked to isolate myself…but you know, now I see that I’m more out-and-about, I’m in contact with lots of other people, I’m in this arena football league.

Fewer veterans endorsed liking the socially connected feature (18/67, 27%). A small subset discussed how this model score increased awareness of a lack of social connection. One veteran stated:

[The app] makes you think if you haven’t gone out with your friends or socialized in a week or two just makes you think “hey, it’s been awhile, maybe I should go out and do something.”

Other veterans discussed the sense of connection and caring they felt with study clinicians who monitored the clinician dashboard. One participant who was the recipient of a clinical outreach during the study expressed:

In other stuff…anytime I reached out before, it was like I was reaching out to a robot... And then, I am using this app instead and I am answering questions and I started getting phone calls and getting help. After I would answer the survey questions from the Companion app and someone would call me to check in on me. At first, I thought that was odd but then I thought that was kinda cool. I actually liked that.

Slightly less than five percent (3/67, 4%) of the veterans reported not liking the app. Moreover, qualitative data suggest that a small subset of participants felt neutral about the app. Many of the participants who expressed neutrality noted its potential utility for different populations, such as those struggling with more severe symptoms of posttraumatic stress disorder, depression, or those at increased risk for suicide. Furthermore, some participants noted the belief that individuals who are more “tech savvy” may reap great benefit.

Very few participants felt that the app violated their personal privacy (4/67, 6%). Nonetheless, qualitatively, a small number of participants shared privacy and monitoring concerns. One veteran stated:

I feel like I’m being monitored, my emotions or how I feel or what I’m doing...when I’m out and about you know like it follow me. I feel like I’m being spied on, but I know I’m not.

A small group of veterans believed that the study team was listening to the recordings, as one veteran noted:

I felt that I had to be careful about what I said or how I said it.

This hesitancy may have had an effect on the frequency and types of messages they recorded. This theme tended to emerge for participants who either expressed a lack of understanding of the app or the technology embedded within the app at the 3-month follow-up visit, or who shared larger concerns regarding privacy and the government on whole

## Discussion

### Principal Findings

The feasibility of implementing the Cogito Companion app and system was evaluated via willingness of veterans to download and use the app; thereby facilitating tracking and outreach by study clinicians. Veterans were willing to enroll in this study, download the app, and keep the app on their phone for the 3-month study period. Participants varied in terms of their interaction with app features. That is, some participants were highly interactive with the app, checking their own model scores, leaving audio check-ins, and completing biweekly surveys. Other participants were not as interactive, leaving audio check-ins or completing surveys only. Another subset had minimal interaction with the app. Overall, we found that the highest active use of the app by veterans occurred in the first 2 weeks, with a decline in interaction over the course of the study. This finding is comparable to other studies that have found that mHealth app use declines over time [24,25]. This pattern has also been seen in mental health interventions, where treatment engagement declines over time [26,27]. Thus, continued investigation into maintaining patient engagement should be considered. Future studies will benefit from examining the integration of gamification techniques or leveraging intrinsic motivation principles to increase the duration of engagement with the app while maintaining successful clinical outcomes.

As noted above, active engagement declined over time; however, clinical tracking of the passive data and biweekly surveys were the unique and important features of this app. Feasibility of daily clinical monitoring was supported. Clinical outreach for approximately 40% (35/83) of the total sample occurred. Of these clinical contacts, one-third (10/35, 29%) were contacted due to changes in the passive data model scores. Frequently, the passive data signaled changes in physical health, psychological functioning or psychosocial stressors (eg, job, relationships, etc). The identification of possible suicide risk via biweekly Patient Health Questionnaire–9 survey responses was another primary reason for clinical contacts.

Acceptability was determined by veteran self-report and qualitative interview at the end of the 3-month study period. Veterans in this study reported overall satisfaction with the Cogito Companion app, demonstrating acceptability. That is, the scores on the Client Satisfaction Questionnaire–8 were above the cutoff indicative of acceptability of the app. At the end of the study about half of the sample (34/67, 51%) reported enjoying the app a lot. Qualitative interviews yielded specific feedback regarding features of the app and level of interaction with the app. Some veterans found completing self-assessments and monitoring their day-to-day symptoms and overall mood to be useful. This is consistent with prior literature that found that patients receiving mental health services were open to monitoring their health via their smartphones [28,29]. Such monitoring may provide an innovative way to improve access to care or to facilitate measurement-based care [30]. Veterans reported finding other app features such as the out-and-about and socially connected scores to be less useful. Research examining which subset of veterans liked or disliked these model scores is needed.

Qualitative feedback provided by the veterans did not indicate a need to significantly modify app features. On the other hand, veterans endorsed wanting a clearer understanding of how the app model scores were obtained. Future studies will benefit from integrating real-time feedback and helpful definitions within the app to properly orient users across the entire duration of app use. Although the study team expected some effect on app use as a result of concerns about privacy, a relatively small subset of the sample expressed these concerns; thereby suggesting that concerns about privacy may not be a limitation for future efficacy trials.

A subset of veterans reported liking a sense of connection to providers conducting outreach calls. Many mHealth apps have primarily passive functions, such as providing psychoeducation and self-management tools and coping strategies. The Cogito Companion app extends these functions by including daily clinical tracking of symptoms and behaviors with the potential for subsequent clinical outreach and intervention, as indicated. Although further evidence is needed, integration of the app and accompanying clinical dashboard into mental health clinics and organizations may be an acceptable way for health care providers to facilitate consistent and caring clinical contacts with their patients.

Even veterans who reported a limited benefit from participation with the app acknowledged the impact such technology may have for at-risk populations. Whereas this study had an inclusive recruitment approach, future studies may wish to study the acceptability, feasibility, and efficacy of mHealth apps such as the Cogito Companion in specific populations. High-risk, chronically health-challenged, or rural-dwelling individuals may derive benefits from such apps. Future investigations of mHealth apps will benefit from clinical trials to establish rigorous and validated mHealth apps among diverse populations.

### Limitations

A few feasibility limitations that were noted were related to technological matters. At the time of this study, the Cogito Companion system was compatible with Android-based smartphones only, which did not permit enrollment of iPhone users. Although it is not expected that differences would be observed between Android-based smartphone and iPhone users, future research may want to ensure that the app is compatible with all smartphone platform types ensuring generalizability. Other limitations included disruption of use due to app upgrades and interruptions in internet connectivity. While technological limitations are expected for any mHealth app, future investigations should account for these issues and take steps to minimize disruptions. Another technological limitation may be the app’s reliance on participant use of text messaging as a necessary variable to populate specific model scores (ie, socially connected and energized). It could be that some veterans do not use SMS text messaging to communicate with family and friends. In fact, a survey on mobile messaging and social media found that 36% of smartphone users communicate via messaging apps separate from SMS text messaging [31]. This same survey report showed that these types of messaging apps are most popular among those aged 18 to 29 years. If veterans in this study used other social media modalities to communicate (such as FaceBook Messenger, SnapChat, WhatsApp, etc), data would not be collected or recorded on the Cogito Companion app nor observed in the clinical dashboard. As a result, passive model score tracking for both the veteran and the clinician may have been less useful and less reflective of daily functioning.

### Conclusions

Feasibility and acceptability of a smartphone app to monitor mental health symptoms, behaviors, and follow-up were generally supported. Veterans reported that the app was generally convenient and easy to use. Benefits of use included an improved sense of self-awareness, motivation to increase activity and engagement, and enhanced sense of connection with care providers. Technological improvements and a reduction of technological barriers are needed to ensure continued use. The use of mHealth apps such as the Cogito Companion app may provide an accessible and portable way for patients to track symptoms or behaviors and share changes with providers.

## Abbreviations

DSM: Diagnostic and Statistical Manual of Mental Disorders
GPS: global positioning system
mHealth: mobile health
